# Sodium Taurocholate Micelles in Fluorometrix Analysis

**DOI:** 10.6028/jres.093.112

**Published:** 1988-06-01

**Authors:** Linda B. McGown, Kasem Nithipatikom

**Affiliations:** Department of Chemistry, Gross Chemical Laboratory, Duke University, Durham, NC 27706

Sodium taurocholate (3*α*,7*α*, 12*α*-trihydroxy-5*β*-cholanoyl taurine, sodium salt), NaTC, is a micelleforming bile salt. The three hydroxy groups on the cholic acid part of the molecule play an important role in the micellar characteristics of NaTC, and, along with the taurine group, serve to solubilize NaTC in aqueous solutions [1]. The most commonly observed micellar form has an aggregation number of four and can bind other molecules in the relatively hydrophobic interior, usually with a 1:1 stoichiometry [2]. The formation of secondary micelles has not been observed. Insoluble biological lipids can be solubilized by comicellization with NaTC [3] and NaTC can also form mixed micelles with detergent molecules [2]. The micellar properties of NaTC are relatively insensitive to experimental conditions. The aggregation number and critical micelle concentration (CMC) show very little dependence on pH in the range of 1.6–10 [1], on counterion concentration, and on temperature in the range of 10–60 °C [3]. A CMC of 3 mM was found from the shift in the absorption wavelength maximum of Rhodamine 6G [3].

We have used fluorescence probe molecules to study NaTC micelles alone and in NaTC-detergent mixed micellar solutions. Results were compared with those obtained for individual detergent micellar solutions, including SDS, Triton X-100 (reduced form) and CTAC. Pyrene was used as a probe of the micellar binding site polarity, and perylene was used in fluorescence lifetime and polarization studies. Nonradiative energy transfer from pyrene to perylene was inhibited in NaTC micellar solutions, and promoted in SDS micelles and SDS-NaTC mixed micelles. Our studies indicate that the binding site characteristics of NaTC micelles, detergent micelles and mixed micelles are all distinctly different from each other.

We have also studied the effects of metal cations, including Eu^3+^, Tb^3+^ and Al^3+^, on the fluorescence intensities of polycyclic aromatic hydrocarbon (PAH) compounds in NaTC micelles. Enhancements as large as 18-fold were observed, and the extent of enhancement tends to increase with decreasing aqueous solubility of the PAH. The fluorescence of relatively soluble PAHs such as phenanthrene and carbazole was quenched by the metal ions in NaTC solutions, suggesting that the more soluble PAHs may not even be bound to the NaTC micelles. Calibration curves were found for several PAHs in NaTC solutions, and were used in the calculation of detection limits for the PAHs which were compared to detection limits obtained in SDS micellar solutions.
